# Transcript Isoforms of SLC7A11-AS1 Are Associated With Varicocele-Related Male Infertility

**DOI:** 10.3389/fgene.2020.01015

**Published:** 2020-09-11

**Authors:** Nafiseh Sanei-Ataabadi, Seyed Javad Mowla, Mohammad Hossein Nasr-Esfahani

**Affiliations:** ^1^Department of Molecular Genetics, Faculty of Biological Sciences, Tarbiat Modares University, Tehran, Iran; ^2^Department of Reproductive Biotechnology, Reproductive Biomedicine Research Center, Royan Institute for Biotechnology, Academic Center for Education, Culture and Research (ACECR), Isfahan, Iran; ^3^Isfahan Fertility and Infertility Center, Isfahan, Iran

**Keywords:** lncRNA, oxidative stress, male infertility, varicocele, SLC7A11-AS1

## Abstract

Oxidative stress is one of the crucial mediators of varicocele-related male infertility. Recently, roles of long noncoding RNAs (lncRNAs) in oxidative stress have begun to emerge, however, little is known about their role in male infertility. The aim of this study was to determine the role of lncRNA SLC7A11-AS1 in varicocele-related male infertility. Through a high-throughput bioinformatics investigation, we predicted that lncRNA SLC7A11-AS1 might be involved in this type of infertility. The reactive oxygen species (ROS) levels and expression levels of SLC7A11-AS1 isoforms were evaluated in ejaculated spermatozoa of 25 infertile men with varicocele and 17 fertile individuals as control. Isoform 6 of SLC7A11-AS1 that showed a significant elevation in infertile men with varicocele relative to the fertile group was overexpressed in testicular-derived carcinoma cell lines (NT2 and NCCIT) followed by assessment of ROS, glutathione (GSH), lipid peroxidation, and cell viability. Overexpression of SLC7A11-AS1 isoform 6 in NT2 and NCCIT cell lines resulted in a significant downregulation of SLC7A11 gene expression, which consequently decreased GSH levels and concomitantly increased ROS levels and enhanced lipid peroxidation, which jeopardized cell survival and promoted cell death. Our finding revealed a potential role of oxidative-related lncRNAs in the pathophysiology of male infertility associated with varicocele.

## Introduction

Globally, infertility affects up to 15% of couples and defined as the failure to conceive after 1-year regular unprotected sexual intercourse (Sharlip et al., 2002). Varicocele is defined as abnormal tortuosity, dilatation, and elongation of the pampiniform plexus veins in the spermatic cord. It is estimated that varicocele accounts for about 35% of male infertility cases; thus, it is considered as a major cause of male infertility. Oxidative stress or excess production of reactive oxygen species (ROS) is a main contributor of the varicocele pathophysiology (Agarwal et al., 2012a). Under heat and hypoxia stress engendered in men with varicocele, ROS is produced in various types of reproductive system cells such as developing germ cells, Leydig cells, and macrophages. Endothelial cells of the dilated pampiniform plexus and main epididymal cells are the other sources of ROS (Barati et al., 2019). A wealth of independent investigations has demonstrated increased levels of oxidative stress factors including free radicals and lipid peroxidation products and decreased levels of total antioxidant capacity (TAC) in varicocele patients compared to the fertile control group (Abd-Elmoaty et al., 2010; Mostafa et al., 2012; Agarwal et al., 2014; Micheli et al., 2016; Gholirad et al., 2017). Heat and hypoxia are the most important inducers of ROS production in varicocele-related male infertility. Hypoxia is explained as the condition of low oxygen pressure in the environment of a particular tissue or organism. Several studies have reported the adverse effects of hypoxia on male infertility and its relationship with sperm count and motility (Verratti et al., 2008; Ghandehari-Alavijeh et al., 2019). In addition to heat and hypoxia stresses, other potential mediators involved in varicocele-related male infertility include hormonal disturbance and back flow of metabolites. However, the exact mechanisms of varicocele-related male infertility have not been elucidated (Agarwal et al., 2012b). Therefore, identification of biological markers related to this type of male infertility is crucial and informative.

Recently, long noncoding RNAs (lncRNAs), i.e., a set of non-protein-coding RNAs with the length of above 200 nucleotides, have been proposed as attractive diagnostic biomarkers and therapeutic targets. This is due to the fact that lncRNAs have tissue-specific expression patterns, relatively easy to detect in various tissues, and eventually are important gene expression regulatory factors in various biological processes. This class of noncoding RNAs regulates its targets in cis (regulates its neighboring protein-coding genes) or trans (regulates distal protein-coding genes) (Cabili et al., 2011). Initially, characterization of lncRNAs in human sperm was performed with RNA sequencing technique by Sendler et al. (2013). Despite the presence of many potential lncRNAs in human sperm, their expression and function in varicocele-related male infertility remain to be determined (Luk et al., 2014). Recently, Zhao et al. (2018) assessed the expression levels and function of an oxidative stress-related lncRNA, named growth-arrested DNA damage-inducible gene 7 (GADD7), in spermatozoa of infertile men with varicocele. They demonstrated that this lncRNA is markedly upregulated in varicocele patients compared to the healthy control group. They believed that regulation of cell proliferation and apoptosis by this lncRNA account for reduced sperm count in varicocele patients (Zhao et al., 2018).

In the present study, we aimed to deduce potential cis-acting lncRNAs in varicocele-related male infertility based on the proximity of sperm-specific lncRNA genes to protein-coding genes of hypoxia, oxidative stress, and male infertility. Using this strategy, we identified several potential cis-acting lncRNAs that we selected, SLC7A11-AS1 among them, for further functional analysis. So, we assessed the expression levels of major isoforms of SLC7A11-AS1 and their relationship with ROS production in spermatozoa samples of infertile men with varicocele using real-time qPCR. Moreover, we measured ROS levels, lipid peroxidation, and cell death after overexpression of two isoforms of SLC7A11-AS1 in NT2 and NCCIT cell lines. Our data revealed a potential causative role for hypoxia and oxidative stress-related lncRNAs and showed their association with excess production of ROS and subsequent impairment of sperm parameters in infertile men with varicocele.

## Materials and Methods

### Long Noncoding RNA Selection Based on the Proximity of Sperm-Specific Long Noncoding RNAs to Coding Genes of Hypoxia and Oxidative Stress Pathways

Firstly, in order to identify neighboring potential cis-acting lncRNAs in infertile men, sperm-specific lncRNAs were implemented from the study by Sendler et al. (2013) on whole RNA-seq data of normal sperm. Then, chromosomal location of each lncRNA was retrieved from UCSC genome browser (GRCh37/hg19) (Kent et al., 2002).

**FIGURE 1:**
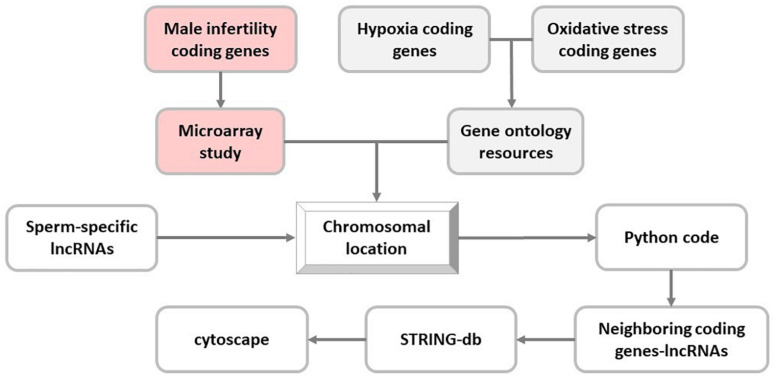
*In silico* flowchart of this study. In this study, long noncoding RNA (lncRNA) selection is based on the proximity of sperm-specific lncRNA to coding genes of hypoxia, oxidative stress, and male infertility pathways.

Next, coding genes related to hypoxia and oxidative stress pathways were extracted from the Gene Ontology Resources (Release: 2019-07-01) (Ashburner et al., 2000; The Gene Ontology Consortium, 2019), and their chromosomal locations were determined in UCSC genome browser (GRCh37/hg19). Moreover, proximity of selected sperm-specific lncRNAs to these genes was analyzed using Python programming language (v3.6.0) (Supplementary File S1). To this end, Python programming language code determined the co-localized sperm-specific lncRNA and stress-related coding genes in the same chromosome with distance less than 50 kb from each other (Figure 1).

### Selection of Long Noncoding RNAs Based on Their Proximity to Protein-Coding Infertility-Related Genes

Differentially expressed mRNAs in spermatozoa of infertile men were obtained from a microarray study consisting of infertile men with idiopathic infertility (normozoospermic patients, *n* = 20) as well as known fertile men (*n* = 20) using Affymetrix GeneChip Human Gene 1.0 ST Array (Bansal et al., 2015). Chromosomal locations of differential expressed genes were determined in UCSC genome browser (GRCh37/hg19). The neighborhood (with a distance of 50 kb) of these male infertility coding genes with sperm-specific lncRNAs was assessed using Python programming language as previously mentioned.

### Network Construction

The protein–protein interaction (PPI) network of protein-coding genes was constructed *via* The Search Tool for the Retrieval of Interacting Genes database (STRING db) (Mering et al., 2003). To this end, protein-coding genes were uploaded to the STRING (v.11.0) online tool with a minimum required interaction score of 0.4. The results of STRING db were visualized by Cytoscape (v.3.7.0) software (Shannon et al., 2003).

### Patients

The present study received the approval of the Royan Ethical Committee (ID. No. IR.ACER.ROYAN.REC.1397.272). Informed written consents were obtained from all participants. The study groups included 25 individuals with grade II or III varicocele referring to Isfahan Fertility and Infertility Center (IFIC) for treatment of infertility and 17 healthy donors with normal semen parameters without any clinical presentation of varicocele referring to IFIC for family balancing. Individuals with azoospermia, leukocytospermia, seminal sperm antibodies, fever within 90 days prior to the seminal analysis, cancer, abnormal hormonal profile, testicular size discrepancy, recurrent varicocele, grade I varicocele, urogenital infections, previous history of scrotal trauma or surgery, drug consumption, excessive alcohol, and occupational exposure to heat were excluded from this study.

### Semen Samples

All semen samples were collected by masturbation into sterile containers after 3–5 days of sexual abstinence and allowed to liquefy at room temperature for 30 min. One portion of the semen was used for semen analysis according to World Health Organization [WHO] (2010) for assessment of sperm concentration, morphology, motility, and vitality. The remaining portion of the semen sample was washed twice in phosphate buffer saline (PBS; pH 7.4) and used for RNA extraction. It is known that semen samples may contain somatic cells and/or germ cells, and their RNA content may have affected our result. Although this is a shortcoming of this study, but we could not use gradient centrifugation as shearing forces induce ROS production.

### Plasmid Construction and Cell Culture and Transfection

Full-length cDNA of SLC7A11-AS1:6 (length: 639 bp) was amplified from normal sperm cDNA using specific primers and subcloned into EcoRI and XhoI sites of pcDNA3.1 to form pcDNA.SLC-AS6. Amplicon product was validated by Sanger sequencing.

Testicular germ cell carcinoma cell lines NTERA2 (NT2) and NCCIT were used in this study. NT2 cells (Royan Institute for Stem Cell Biology and Technology, ACECR, Iran, Tehran) were grown in Dulbecco’s modified Eagle’s medium (DMEM) containing 10% fetal bovine serum (FBS), 1% L-glutamine, 1% non-essential amino acid (NEAA), and 1% penicillin/streptomycin. NCCIT cells (Pasteur Institute, Iran, Tehran) were cultured in RPMI-1640 medium supplemented with 10% FBS and 1% penicillin/streptomycin. Both cell lines were incubated at 37°C and 5% CO_2_. The day before transfection, cells were seeded in 12-well plate with a density of 2 × 10^5^ cell per well. Transfection of NT2 and NCCIT cells were performed using lipofectamin LTX Reagents (Invitrogen, United States), according to the manufacturer’s instructions.

### Assessment of Reactive Oxygen Species Production Using 2′,7′-Dichlorodihydrofluorescein Diacetate Probes

2′,7′-Dichlorodihydrofluorescein diacetate (DCFH-DA) (Sigma-Aldrich, MO, United States), a cell-permeable stain, was used for assessment of ROS produced by NT2 and NCCIT cells and spermatozoa samples. DCFH-DA (0.5 μM) was added to the NT2/NCCIT cells and sperm suspension (1 × 10^6^ sperm ml^–1^ in PBS), incubated at 37°C for 30 min, and subsequently analyzed using FACSCalibur flow cytometer (Becton Dickinson, San Jose, CA, United States). Green fluorescent was evaluated by the fluorescence detector-1 (FL-1) with a 530/30 nm band pass filter. Raw data were analyzed using CellQuest Pro and WinMDI 2.9 software. Unstained control sample was used for normalization of the corresponding test sample. Data were expressed as the percentage of fluorescent positive NT2/NCCIT or spermatozoa cells.

### RNA Extraction and cDNA Synthesis

Total RNA was extracted from sperm pellets and transfected to NT2/NCCIT cells using TRIzol reagent (Invitrogen, United States) according to the manufacturer’s supplied instructions. In order to eliminate possible genomic DNA contamination, total RNA was treated with RNase-free DNaseI (Thermo Fisher Scientific, United States). Synthesis of first-strand cDNA was carried out using PrimeScript II 1st strand cDNA synthesis kit (TaKaRa, Japan).

### Real-Time qPCR

RT-qPCR was performed in Step One Plus Real-Time PCR thermal cycler (Applied Biosystems, United States) using SYBR Premix Ex TaqII (TaKaRa, Japan). All reactions were carried out in triplicate using specific primers. PCR was implemented during three steps with an initial denaturation at 95°C for 30 s and 40 cycles consisting of a denaturation step at 95°C for 5 s, an annealing step at specific annealing temperature for 10 s, followed by an extension step for 30 s at 72°C. Real-time specific primers were presented in Supplementary Table S1. The expression levels of lncRNAs were normalized by expression of the housekeeping gene glyceraldehyde 3-phosphate dehydrogenase (GAPDH). The calculation of relative expression was assessed using the ΔΔCt method (Livak and Schmittgen, 2001; Vaerman et al., 2004).

### Western Blotting

Total protein was extracted using TRIzol reagent (Invitrogen, United States) according to the manufacturer’s protocol. Equal amounts of each sample protein (30 μg) were separated on sodium dodecyl sulfate-polyacrylamide gel electrophoresis (SDS–PAGE) followed by transferring onto a polyvinylidene fluoride (PVDF) membrane. The skim milk-blocked membranes were probed with the following antibodies: GAPDH (Millipore; mab374; 1:5,000) and SLC7A11 (Abcam; ab37185; 1:200) as primary antibodies; horseradish peroxidase (HRP)-conjugated goat anti-mouse immunoglobulin G (IgG) (Dako; P0447; 1:5,000) and HRP-conjugated goat anti-rabbit IgG (Santa Cruz; SC2301; 1:16,000) as secondary antibodies. Finally, ECL Advance Western blotting Detection Kit (Amersham Biosciences, RPN2135) was used for visualization of HRP-conjugated IgG bound to each protein band.

### Glutathione Assay

Levels of the reduced form of glutathione (GSH) were determined using colorimetric method. This method is based on oxidation of glutathione by DTNB [sulfhydryl reagent 5,5′-dithio-bis(2-nitrobenzoic acid)] to produce TNB (5′-thio-2-nitrobenzoic acid) chromophore that has maximal absorbance at 412 nm (Ellman, 1959; Rahman et al., 2006). Briefly, 48 h after transfection, 1 × 10^6^ cells were resuspended in 10% tricolor acetic acid (TCA) and homogenized through sonication. Then, 0.1 ml supernatant was mixed with DTNB in 0.1 M potassium phosphate buffer with 5 mM ethylenediaminetetraacetic acid (EDTA), and its absorbance was monitored spectrophotometrically at 412 nm. Intracellular GSH content was measured based on GSH standard curve.

### Assessment of Lipid Peroxidation

Lipid peroxidation levels were determined by monitoring the shift of the C11-BODIPY 581/591 (D3861, Molecular Probes) fluorescence emission from red to green. Cells 1 × 10^6^ were incubated with C11-BODIPY at final concentration of 3 mM for 30 min at 37°C followed by twice washing with PBS. The percentage of C11-BODIPY-positive cells was assessed using a FACSCalibur flow cytometer (Becton Dickinson, San Jose, CA, United States).

### Cell Death Analysis

Cells undergoing apoptosis were detected using propidium iodide (PI) and annexin V-FITC (IQ Products, IQP-120F) 48 h after transfection of NT2 and NCCIT cells in 12 well plates, following the manufacturer’s instruction.

### Cell Proliferation Assay

Cell proliferation was measured using MTS [3-(4,5-dimethylthiazol-2-yl)-5-(3-carboxymethoxyphenyl)-2-(4-sulfophenyl)-2H-tetrazolium] assay. Cells were seeded and transfected in 96 well plates. After 0, 24, 48, and 72 h from transfection, cells were incubated in 10% MTS reagent (Promega, Madison, WI, United States) for 4 h at 37°C. Subsequently, the absorbance of each well was measured at 492 nm using a spectrophotometric plate reader (Stat Fax 3200).

### Statistical Analysis

Data normality was assessed using Kolmogorov–Smirnov *Z*-test. Statistical analysis was performed by independent samples *t*-test, and values are represented as mean ± SEM. Pearson correlation coefficient was used for correlation analysis. GraphPad Prism (version 8.2.0; GraphPad software, California Corporation, CA, United States) and SPSS 20 (SPSS, Chicago, IL) software were used for statistical analyses. For all analyses, *p* < 0.05 were considered statistically significant and indicated with a star in the columns.

## Results

### Putative Long Noncoding RNA With Proximity to Infertility-Related Coding Genes

Since hypoxia and oxidative stress are two important potential mechanisms of varicocele-related male infertility, our criteria for selection of putative lncRNAs in varicocele-related male infertility was based on co-localization of sperm-specific lncRNAs with hypoxia, oxidative stress, and male infertility protein-coding genes.

We selected 83 sperm-specific lncRNAs, including 154 transcripts from previous RNA sequencing data on normal sperm (Supplementary Table S2).

**FIGURE 2:**
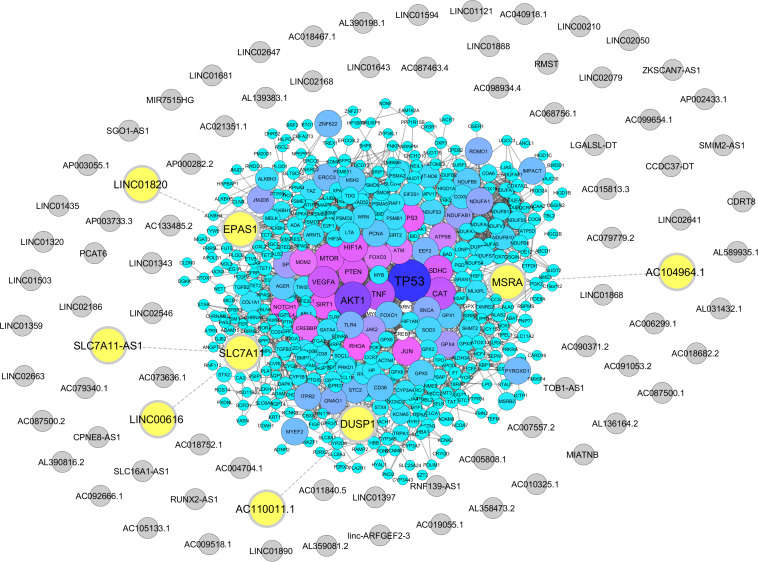
Association of hypoxia and oxidative stress coding genes with sperm-specific long noncoding RNAs (lncRNAs). Protein-coding genes related to hypoxia and oxidative stress were retrieved from the Gene Ontology Resource, and their neighboring sperm-specific lncRNAs were assessed by Python programming language, transferred to STRING database, and visualized *via* Cytoscape. The protein-coding genes are represented by circles with a color range from blue to purple. Sperm-specific lncRNAs are indicated by gray circles. Neighboring lncRNA–coding gene pairs are indicated by bolded yellow circles.

From Gene Ontology resources, we identified 581 and 358 coding genes involved in oxidative stress and hypoxia pathways, respectively (Supplementary Tables S3, S4). Out of these two lists, we found five lncRNAs neighboring to four hypoxia and oxidative stress coding genes with a distance less than 50 kb. These lncRNAs including AC104964.1, SLC7A11-AS1, LINC00616, LINC01820, and AC110011.1 are located in the proximity of protein-coding genes *MSRA, SLC7A11, EPAS1*, and *DUSP1* (Figure 2 and Supplementary Tables S5, S6).

**FIGURE 3:**
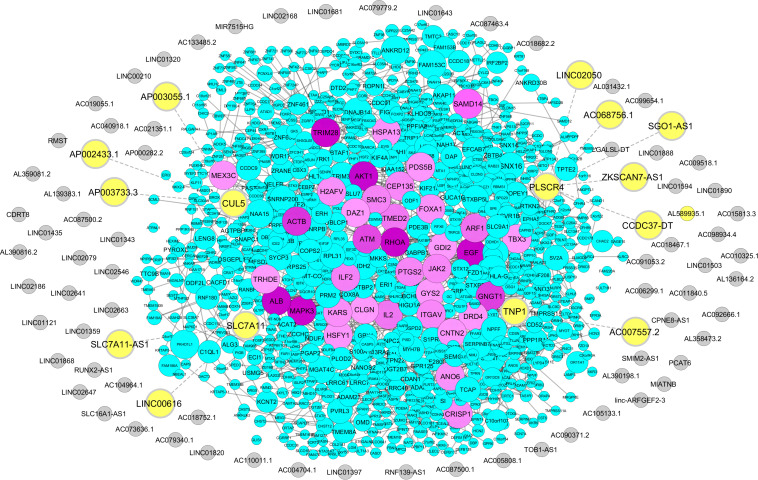
Association of male infertility-related coding genes with sperm-specific long noncoding RNAs (lncRNAs). Male infertility-related coding genes were obtained from the microarray study, and their proximity to sperm-specific lncRNAs was examined *via* Python programming language, uploaded to STRING database, and visualized *via* Cytoscape. The protein-coding genes are indicated by circles with a color range from blue to purple. Sperm-specific lncRNAs are represented by gray circles. Neighboring lncRNA–coding gene pairs are indicated by bolded yellow circles.

We retrieved 1,768 coding genes from a microarray study of male infertility-related genes that compared gene expression profiles between idiopathic infertile men and a fertile group (Bansal et al., 2015; Supplementary Table S7). From this list, we identified 11 lncRNAs in proximity of four male infertility coding genes. These lncRNAs include ZKSCAN7-AS1, CCDC37-DT, SGO1-AS1, AC068756.1, LINC02050, AC007557.2, SLC7A11-AS1, LINC00616, AP002433.1, AP003733.3, and AP003055.1. Neighboring genes to these lncRNAs were defined as *PLSCR4, TNP1, SLC7A11*, and *CUL5* (Figure 3 and Supplementary Table S8). Interestingly, SLC7A11-AS1 was common between two approaches of stress and male infertility list, so it was chosen for further analysis.

### Semen Analysis

The results of semen analyses of varicocele and fertile groups are presented in Table 1. No differences were noted in the mean age and volume between the two groups. The mean sperm count and percentage of sperm motility were significantly decreased while the percentage of abnormal sperm morphology was significantly increased in infertile men with varicocele compared to fertile men, indicating impaired spermatogenesis in the varicocele group.

**TABLE 1:** Descriptive semen parameters.

	Healthy		
Parameters	control (*n* = 17)	Varicocele (*n* = 25)	*P*-value
Age (year ± SD)	35/12 ± 1/286	32/80 ± 0/7874	0.1117
Volume (ml ± SEM)	3.924 ± 0.3088	3.528 ± 0.2616	0.3368
Concentration	90/88 ± 6/741	42/40 ± 4/171	<0.0001
(10^6^/ml ± SEM)			
Motility (% ± SEM)	59/41 ± 2/006	40/56 ± 3/064	<0.0001
Abnormal sperm	95.41 ± 0.1500	97.40 ± 0.2582	0.0037
morphology (% ± SEM)			

*SD, standard deviation; SEM, standard error of mean.*

### Among Various SLC7A11-AS1 Isoforms, SLC-AS6 and SLC-AS7 Are Associated With Varicocele-Related Male Infertility

First of all, ROS levels were assessed in spermatozoa of varicocele and fertile groups using DCFH-DA staining. The percentage of DCF-positive sperm was significantly elevated in infertile men with varicocele compared to fertile men, indicating high levels of oxidative stress in the varicocele state. In addition, the correlation between percentages of total DCF-positive sperm with semen parameters was assessed and showed significant negative correlations with sperm count and motility (Supplementary Figure S1).

**FIGURE 4:**
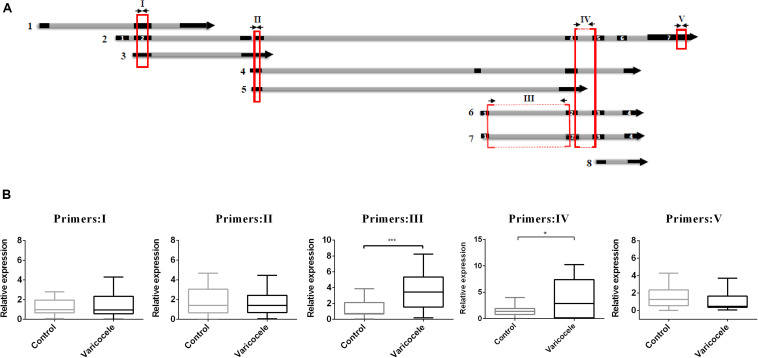
Comparison of relative expression levels of various SLC7A11-AS1 isoforms in spermatozoa of individuals with varicocele with those of fertile men. **(A)** Schematic representation from eight isoforms of SLC7A11-AS1. Five primer pairs (I–V) were used for evaluation of expression in spermatozoa samples (primers were indicated by arrows; qPCR-amplified products were indicated by red boxes). **(B)** Quantitative RT-PCR analysis of the expression of SLC7A11-AS1 isoforms using five primer pairs in the ejaculated spermatozoa of varicocele and control groups. Glyceraldehyde 3-phosphate dehydrogenase (GAPDH) was used for expression normalization. *P < 0.05 and ***p < 0.001 using independent sample *t*-test.

When we decided to determine the role of SLC7A11-AS1 in varicocele-related male infertility, we noticed that this lncRNA had eight different isoforms (LNCipedia v. 5.2)^1^. We evaluated the expression levels of major isoforms using five overlapping or specific primer pairs (primer I: isoforms 1, 2, 3; primer II: isoforms 2, 3, 4, 5; primer III: isoforms 2, 6, 7; primer IV: isoforms 6, 7; primer V: isoform 2) (Figure 4A). We found a significant increase in expression levels when we used primer pairs III and IV. So we deduced that the maximum significant expression belongs to isoforms SLC7A11-AS1:6 (SLC-AS6) and SLC7A11-AS1:7 (SLC-AS7) in spermatozoa of the varicocele patients (Figure 4B). It is important to note that this deduction was based on similar primer efficiencies between primer pairs.

**FIGURE 5:**
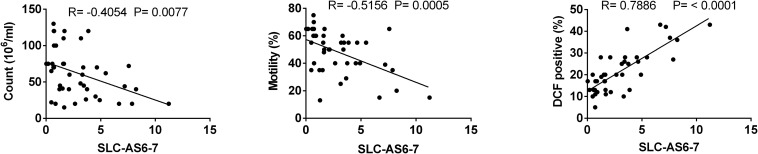
Correlation analysis between expression levels of isoforms SLC-AS6 and SLC-AS7 (SLC-AS6-7) with sperm count, motility, and reactive oxygen species (ROS) levels. Pearson’s coefficient correlation was implicated for correlation analysis.

To assess whether SLC-AS6 and SLC-AS7 specifically participate in varicocele-related male infertility, we examined the correlation of expression level of all isoforms with ROS and sperm parameters. As expected, only SLC-AS6 and SLC-AS7 showed a significantly positive correlation with ROS levels and negative correlation with sperm count and motility in our spermatozoa samples. It is notable that there is more than 95% identity between the sequence of SLC-AS6 and SLC-AS7, so we preferred to evaluate their expression levels using common primers and indicated them as SLC-AS6-7 (Figure 5).

### Regulation of SLC7A11 by SLC-AS6

**FIGURE 6:**
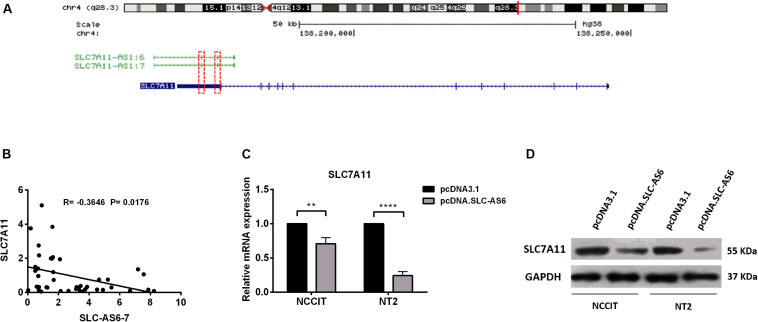
Regulation of SLC7A11 by SLC-AS6-7. **(A)** Chromosomal organization of SLC7A11 and two SLC7A11-AS1 isoforms, SLC7A11-AS1:6 (SLC-AS6) and SLC7A11-AS1:7 (SLC-AS7). Exons are illustrated as solid boxed lines, whereas introns are represented as barbed lines indicating the direction of transcription. Red dashed boxes indicated overlapping complementary regions between SLC7A11 and SLC-AS6-7. **(B)** The expression levels of SLC-AS6-7 negatively correlated with SLC7A11. Pearson’s coefficient correlation was implicated for correlation analysis. **(C)** When SLC-AS6 was overexpressed in NT2 and NCCIT cells, significant downregulation of SLC7A11expression levels was detected by qRT-PCR. Error bars show standard error of mean (SEM) of triplicated experiments (**P < 0.01 and ****P < 0.0001 using independent sample *t*-test). **(D)** Overexpression of SLC-AS6 leads to downregulation of SLC7A11 protein level in NT2 and NCCIT cells. Cropped images were used for Western blot, and uncropped images are presented in Supplementary Figure S3.

*In silico* studies revealed that there is a natural antisense transcript, SLC7A11, in the vicinity of SLC7A11-AS1 gene. Moreover, there is an overlapping complementary sequence region between SLC7A11 and SLC-AS6-7 at chromosome 4q28.3 locus (Figure 6A). In order to evaluate the potential cis-regulatory role of SLC-AS6 and SLC-AS7 in varicocele-related male infertility, we examined the correlation between expression levels of SLC-AS6-7 and neighboring sense transcript, SLC7A11, that showed a significant negative correlation in spermatozoa samples (Figure 6B).

Hence, we aimed to overexpress SLC-AS6 as a representative of these two isoforms in testicular germ cell carcinoma cell lines NT2 and NCCIT (Supplementary Figure S2). Surprisingly, 48 h post-transfection of SLC-AS6, a significant downregulation of neighboring sense SLC7A11 was detected by qPCR (Figure 6C). Western blot analysis also confirmed a decreased expression of SLC7A11 after transfection of SLC-AS6 in NT2 and NCCIT cell lines (Figure 6D). Altogether, our data suggested that SLC7A11 is likely to be targeted by SLC-AS6.

### Oxidative Stress Markers Were Significantly Changed in Response to SLC-AS6

**FIGURE 7:**
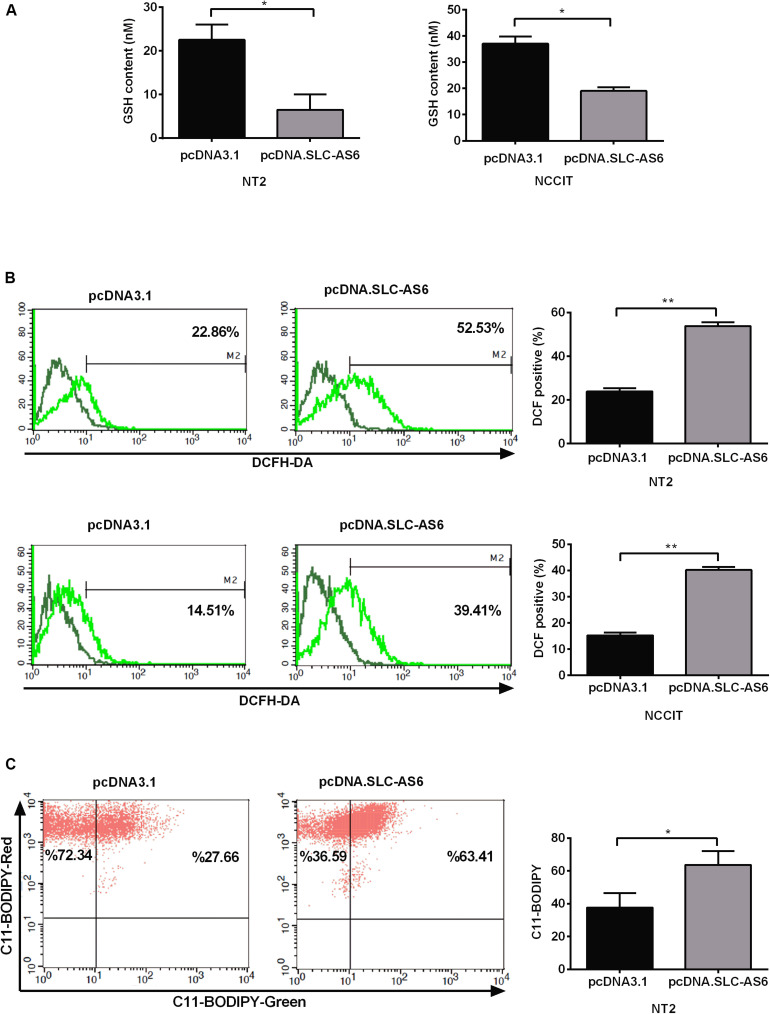
Assessment of oxidative stress markers after overexpression of SLC-AS6. **(A)** Transfecting SLC-AS6 diminished the glutathione (GSH) levels significantly relative to mock-transfected cells in NT2 and NCCIT cell lines. **(B)** Cytosolic reactive oxygen species (ROS) levels were elevated significantly after overexpression of SLC-AS6 in NT2 and NCCIT cells. **(C)** Transfecting SLC-AS6 in NT2 cell line leads to an increase in lipid peroxidation detected by C11-BODIPY staining. Error bars show standard error of mean (SEM) of triplicated experiments (*P < 0.05 and **P < 0.01 using independent sample *t*-test).

SLC7A11 is a light-chain subunit of xCT cystine/glutamate transporter that acts as the most important rate-limiting factor for intracellular cysteine. Since cysteine is a critical precursor of GSH, we aimed to asses GSH contents of transfected NT2 and NCCIT cells with SLC-AS-6. Our results demonstrated that GSH contents significantly diminished in NT2 and NCCIT cell lines transfected with SLC-AS-6 (Figure 7A). Since GSH is the main cellular defense barrier against oxidative stress, we examined cytosolic ROS 48 h after overexpression of SLC-AS6 in NT2 and NCCIT cells using the fluorescent probe H2DCFDA. Cytosolic ROS levels were significantly increased in NT2 and NCCIT cell lines after overexpression of SLC-AS6 compared to control (Figure 7B).

One of the most important consequence of excess production of ROS in cells is lipid peroxidation, wherein ROS react with polyunsaturated fatty acids (PUFAs) of the cellular membrane, which leads to the production of reactive intermediates. Therefore, we evaluated lipid peroxidation after overexpression of SLC-AS6 by C11-BODIPY staining in NT2 cell line. The results showed a significant enhancement of lipid peroxidation after overexpression of SLC-AS6 (Figure 7C).

### Overexpression of SLC-AS6 Altered the Survival of Cell Lines

**FIGURE 8:**
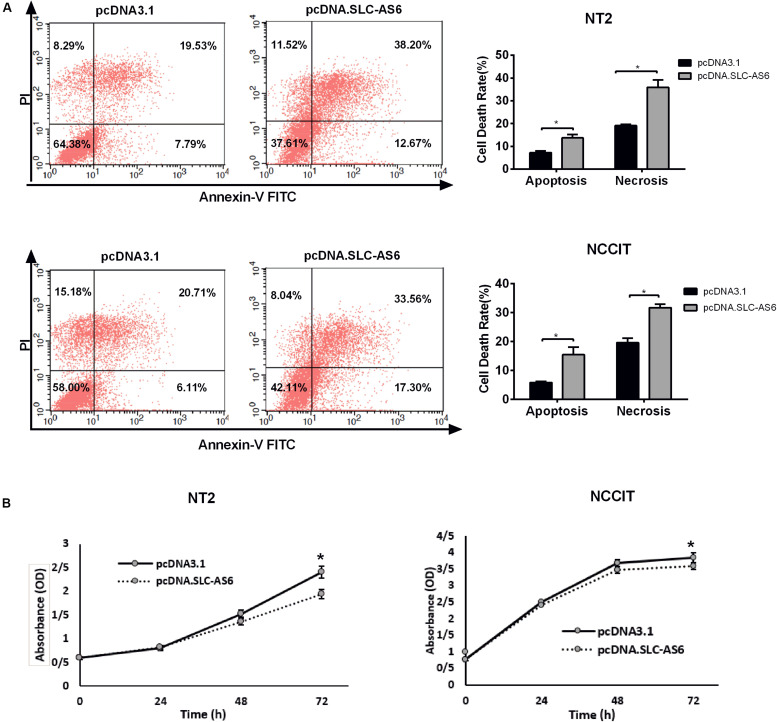
Effect of SLC-AS6 overexpression on cell viability. **(A)** Percentages of apoptotic (Annexin-positive, PI-negative) and necrotic cells (Annexin- and PI-positive) were elevated significantly in NT2 and NCCIT cell lines transfected with SLC-AS6. **(B)** MTS [3-(4,5-dimethylthiazol-2-yl)-5-(3-carboxymethoxyphenyl)- 2-(4-sulfophenyl)-2H-tetrazolium] assay results showed that overexpression of SLC-AS6 significantly decreased survival rate of NT2 and NCCIT cells lines. Error bars show standard error of mean (SEM) of triplicated experiments (*P < 0.05 using independent sample *t*-test).

To further explore the effect of SLC-AS6 overexpression on cell viability, we evaluated cell death in transfected NT2 and NCCIT with SLC-AS6 by annexin V-PI assay. Percentages of cells undergoing apoptosis (Annexin-positive, PI-negative) and necrosis (Annexin- and PI-positive) were increased significantly in response to SLC-AS6 compared with mock-transfected cells (Figure 8A). Moreover, MTS assay supported the effects of SLC-AS6 overexpression in significant survival reduction in NT2 and NCCIT cell lines (Figure 8B). These results demonstrated that excess production of ROS due to decreased GSH content promotes cell death including apoptosis and necrosis when SLC-AS6 was overexpressed in NT2 and NCCIT cells.

## Discussion

It is well accepted that varicocele is one of the most important causes of male infertility through induction of hyperthermia, oxidative stress, and hypoxia. However, the exact molecular pathological processes remain to be elucidated.

Growing evidence has demonstrated the crucial roles of ncRNAs in the regulation of hypoxia and oxidative stress, two main elements contributing to varicocele-related male infertility (Kilinc et al., 2004; Paick et al., 2012; Giannakakis et al., 2015; Wang et al., 2016; Shih and Kung, 2017).

Previous studies have identified stress-related ncRNAs including micro RNAs (miRNAs) and lncRNAs as potential biomarkers for varicocele. MiR-15a, miR-122, miR-181a, and miR-34c5 are stress-related miRNA that are found to be potential mediators in male infertility caused by varicocele (Ji et al., 2014; Mostafa et al., 2016). GADD7 is the first and the only identified stress-induced lncRNA that is involved in the pathophysiology of varicocele-related male infertility (Zhao et al., 2018).

In the present study, we have found lncRNAs with potential involvement in varicocele-related male infertility pathogenesis by applying an approach that was based on identification of lncRNAs with specific expression in sperm that are located in the neighborhood of protein-coding genes involved in hypoxia, oxidative stress, and male infertility pathway. This strategy introduces five lncRNAs in the vicinity of hypoxia and oxidative stress protein-coding genes and 11 lncRNAs in the neighboring male infertility-coding genes. These lncRNAs have great opportunities for association with pathogenesis of varicocele-related male infertility because of their physical association with its key protein-coding genes. Out of these two lists, we selected SLC7A11-AS1 that had unique expression in sperm and was common in both oxidative stress and male infertility pathways.

Recently, SLC7A11-AS1 was evaluated in cancer studies, and their results revealed its association with cancer progression (Luo et al., 2017; Yuan et al., 2017). Since lncRNAs have multiple isoforms that differentially express in various cells/tissues (Zhang et al., 2014; Chowdhury et al., 2017), in the present study, we evaluated the expression levels of several isoforms of SLC7A11-AS1 to identify which isoforms are involved in varicocele-related male infertility. Our results revealed that nearly all SLC7A11-AS1 transcript isoforms are present in spermatozoa samples, but only the expression levels of isoforms 6 and 7 (SLC-AS6-7) showed significant correlations with ROS levels, sperm count, and motility.

Although the presence of somatic cells in semen samples may cause deviation in our gene expressions, in this study, we selected semen samples with the lowest white blood cell (WBC) contents (all semen samples contained < 1 million/ml WBC) to reduce deviation in the gene expression patterns.

Since the role of SLC7A11-AS1 was so far not evaluated in relation to oxidative stress, we used the advantage of NT2 and NCCIT cell lines to assess the function of SLC7A11-AS1 in the regulation of its neighboring protein-coding gene, *SLC7A11*. It is noteworthy that these two cell lines (NT2 and NCCIT) as testicular germ cell carcinoma cell lines have been previously used for functional analysis of lncRNAs in male infertility (Lü et al., 2015; Su et al., 2019).

Notably, overexpression of SLC-AS6 isoform induced a significant decrease in expression levels of SLC7A11. These results suggested that downregulation of SLC7A11 by SLC-AS6 may be through overlapping complementary sequence region between exon 12 of SLC7A11 and two exons (exons 2 and 3) from SLC-AS6. However, further studies are needed to approve this finding.

SLC7A11 (also known as xCT) and SLC3A2 (4F2hc) are two subunits of cystine/glutamate antiporter system Xc- that has a pivotal role in maintaining redox homeostasis. System Xc- imports cystine into the cells in exchange for glutamate (Lewerenz et al., 2013). The entered cystine is quickly reduced to cysteine that serves as a crucial precursor for GSH, the major antioxidant in cells. GSH is the main cofactor of ROS-detoxifying enzymes, such as glutathione peroxidase (GPX), which protect the cells against oxidative stress damage (Koppula et al., 2018).

**FIGURE 9:**
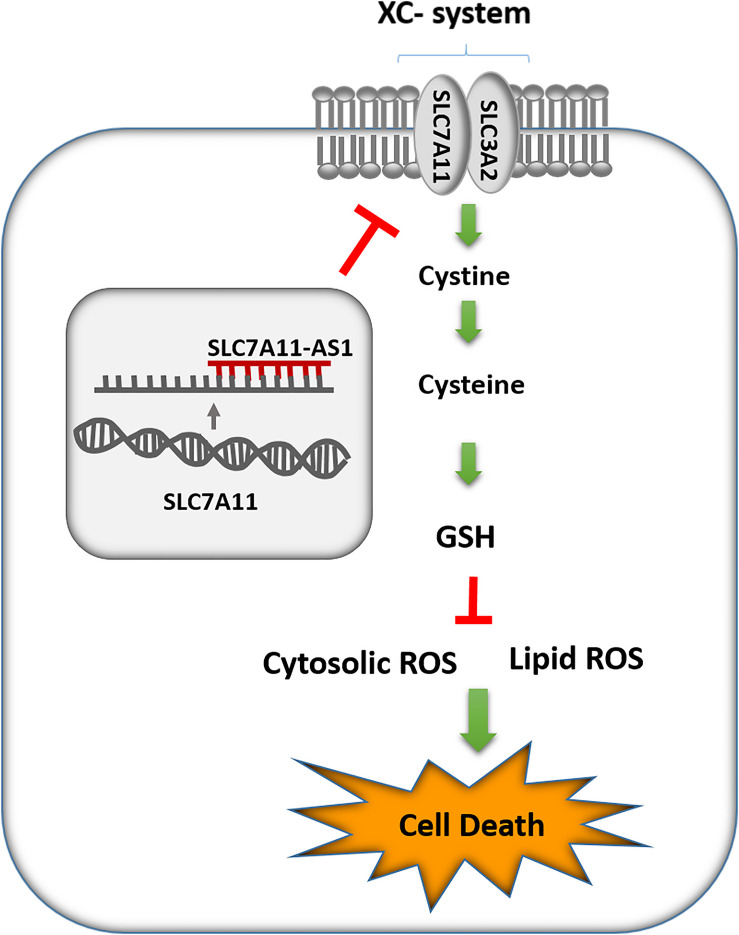
Schematic representation of SLC7A11-AS1 effect on glutathione (GSH) levels, reactive oxygen species (ROS), lipid peroxidation, and cell death.

ROS are by-products of cellular metabolism that can be accumulated mainly by decreasing the efficiency of antioxidant systems such as GSH in cells that results in oxidative stress. Moreover, lipid peroxidation of PUFAs is a consequence of oxidative stress, which in turn can lead to changes in the membrane integrity and finally lead to cell death in various cells specially sperm (Higdon et al., 2012). Notably, sperm cells are more susceptible to oxidative stress because of the abundance of PUFAs in their plasma membranes and low levels of antioxidant enzymes in their cytoplasm (Aitken et al., 2013). In the present study, we found a significant decrease in GSH levels after overexpression of SLC-AS6 in NT2 and NCCIT cell lines, which could be a consequence of SLC7A11-mediated cysteine depletion. Furthermore, we indicated an enhancement in ROS and lipid peroxidation in these cell lines, which might be results of GSH reduction. Finally, we revealed significant elevation in cell death, including apoptosis and necrosis in these cell lines (Figure 9). In recent years, ferroptosis as a programming nonapoptotic cell death was found to be associated with accumulation of lipid ROS through an iron-dependent manner. This new form of cell death is triggered by SLC7A11-mediated cysteine depletion or GPX4 inhibition (Stockwell et al., 2017). Although we did not examine it, we suggest that ferroptosis might be another type of cell death promoted by overexpression of SLC-AS6. However, this hypothesis remains to be elucidated. Our results suggested that SLC-AS6 may serve a potential role in redox homeostasis of human spermatozoa and its upregulation may have a relationship with downregulation of SLC7A11 and subsequent increase of ROS levels that lead to the poor semen parameters in infertile men with varicocele. Recent studies demonstrated novel molecular mediators such as chronic reductive stress, mitochondrial dysfunction, and downregulation of heat shock proteins (HSPs) involved in sperm dysfunction in infertile men with varicocele (Samanta et al., 2018; Swain et al., 2020). Evaluation of the relationship of SLC7A11-AS1 with these mediators in varicocele-related male infertility is suggested for further studies that could elucidate the exact mechanisms in this issue.

Altogether, in this study, we identified several lncRNAs with potential involvement in varicocele-related male infertility based on proximity to hypoxia, oxidative stress, and male infertility-related coding genes. Next, we picked up SLC7A11-AS1 for functional analysis and revealed the potential role of SLC-AS6 in the regulation of SLC7A11, GSH levels, ROS levels, lipid peroxidation, and subsequent cell death in NT2 and NCCIT cell lines. Finally, we found a significant relationship between SLC-AS6 and varicocele-related male infertility. Our study may help to improve our understanding of pathophysiology mechanisms of varicocele-related male infertility and provides new diagnostic biomarkers and therapeutic targets for male infertility.

## Data Availability Statement

All datasets generated for this study are included in the article/Supplementary Material.

## Ethics Statement

The studies involving human participants were reviewed and approved by the Royan Ethical Committee. The patients/participants provided their written informed consent to participate in this study.

## Author Contributions

NS-A contributed to experiment design, acquisition of data or analysis and interpretation of data, and drafting the manuscript. MN-E contributed to experiment design, interpretation of data, revising the manuscript, and giving final approval of the version to be published. SM contributed to experiment design, interpretation of data, revising the manuscript, and giving final approval of the version to be published.

## Conflict of Interest

The authors declare that the research was conducted in the absence of any commercial or financial relationships that could be construed as a potential conflict of interest.

## Footnotes

1 https://lncipedia.org/

## Abbreviations

SLC7A11-AS1:6: SLC-AS6
SLC7A11-AS1:7: SLC-AS7
SLC7A11-AS1:6 and SLC7A11-AS1:7: SLC-AS6-7.
