# Supplementary material for: Transcript Isoforms of SLC7A11-AS1 Are Associated With Varicocele-Related Male Infertility
Source: Front Genet. 2020 Sep 11;11:1015. doi: 10.3389/fgene.2020.01015 (PMC7516207; doi:10.3389/fgene.2020.01015)
Supplement: Supplementary file 1 [file Data_Sheet_1.docx]

File S1: Python's scripts

gene=open("C:\\Users\\Aref\\Desktop\\newgenes.txt","r") lnc=open("C:\\Users\\Aref\\Desktop\\lnc.txt","r")

distance=50000

res=open("C:\\Users\\Aref\\Desktop\\result.txt","w")

for g in range(1,117):

gline=gene.readline()

import string

gcht1=str.find(gline,"\t") # first TAB character

gcrn=int(gline[:gcht1]) #choromosomal number

gcht2=str.find(gline,"\t",gcht1+1) # second TAB character

gfn=int(gline[gcht1+1:gcht2])#first nucleotide in gline

gln=int(gline[gcht2+1:])#last nucleotide in gline

lnc=open("C:\\Users\\Aref\\Desktop\\lnc.txt","r")

for l in range(1,27):

lline=lnc.readline()

import string

lcht1=str.find(lline,"\t") # first TAB character

lcrn=int(lline[:lcht1]) #choromosomal number

lcht2=str.find(lline,"\t",lcht1+1) # second TAB character

lfn=int(lline[lcht1+1:lcht2])#first nucleotide in lline

lln=int(lline[lcht2+1:])#last nucleotide in lline

if gcrn==lcrn:

if abs(gfn-lln)<=distance or abs(lfn-gln)<=distance:

res.write(("%-2d\t%-2d\n")%(g,l))

if gfn>lfn and gfnlfn and gln <lln:

res.write(("%-2d\t%-2d\n")%(g,l))

if gln>lfn and gln <lln:

res.write(("%-2d\t%-2d\n")%(g,l))

res.write("\n")

res.close()

gene.close()

lnc.close()
