# Supplementary material for: Transcript Isoforms of SLC7A11-AS1 Are Associated With Varicocele-Related Male Infertility
Source: Front Genet. 2020 Sep 11;11:1015. doi: 10.3389/fgene.2020.01015 (PMC7516207; doi:10.3389/fgene.2020.01015)
Supplement: Supplementary file 2 [file Data_Sheet_2.zip › Suppl. Table 1.DOCX]

| **Table S1** The list of primer pairs used in this study | | | |
| --- | --- | --- | --- |
| Primer | Sequence (5ˊ-3ˊ) | Tm (˚C) | GC% |
| F-SLC7A11-AS1(I) | ACTGTTCGTCTATCTATATCGTC | 55 | 39.1 |
| R-SLC7A11-AS1(I) | AGTGTTTACAATGCTCGTG | 54 | 42.1 |
| F-SLC7A11-AS1(II) | GGAGAAGAAAGAGACACAGATTAG | 56 | 41.7 |
| R-SLC7A11-AS1(II) | CCCTCAATGGATAGGACAATTC | 56 | 45.5 |
| F-SLC7A11-AS1(III) | CTTTTAAGGAAATCAATATATCACGA | 52 | 26.9 |
| R-SLC7A11-AS1(III) | CATTTAGACTGTAGCTCCTC | 52 | 45.0 |
| F-SLC7A11-AS1(IV) | CTAGGACAAATCTTTACTCTTC | 51 | 36.4 |
| R-SLC7A11-AS1(IV) | TATGTGGTCATTCTCTACAAC | 51 | 38.1 |
| F-SLC7A11-AS1 (V) | TAGAAGTCCAGCAGATGT | 53 | 44.4 |
| R-SLC7A11-AS1(V) | CCAAGTCCAGATGAGAAC | 52 | 50 |
| F-SLC7A11 | TGTCTCCAGGTTATTCTATGTTG | 54 | 39.1 |
| R-SLC7A11 | CCAGAGAAGAGCATTATCATTG | 55 | 40.9 |
| F-GAPDH | CCACTCCTCCACCTTTGACG | 60 | 60.0 |
| R-GAPDH | CCACCACCCTGTTGCTGTAG | 62 | 60.0 |
| F: forward primer; R: reverse primer; Tm: melting temperature. | | | |
