# Supplementary material for: Transcript Isoforms of SLC7A11-AS1 Are Associated With Varicocele-Related Male Infertility
Source: Front Genet. 2020 Sep 11;11:1015. doi: 10.3389/fgene.2020.01015 (PMC7516207; doi:10.3389/fgene.2020.01015)
Supplement: Supplementary file 3 [file Data_Sheet_3.docx]

**Figure S1:** Determination of ROS levels in varicocele and control groups and its relationship with sperm count and motility. **(A)** The ROS levels in varicocele and control groups assessed using DCF-DA staining. **(B)** The ROS levels were negatively correlated with sperm count. **(C)** The ROS levels showed negative correlation with sperm motility.





**Figure S2:** Overexpression of SLC-AS6. Transfecting SLC-AS6 caused in ~ 6000-fold elevation of its expression in NT2 cell line and a ~3000 fold increase in NCCIT cell line (**P<0.01).


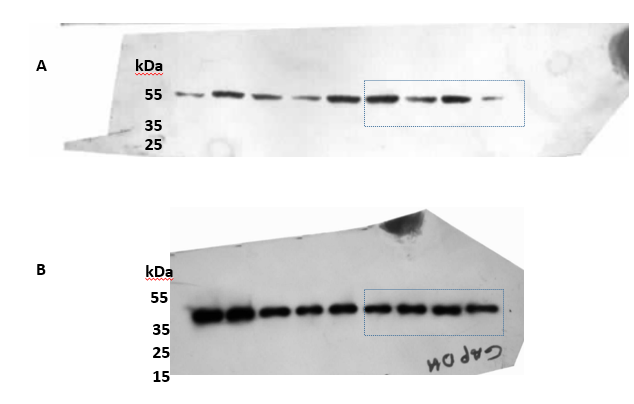


**Figure S3:** Uncropped western blot images. (A) Uncropped and full size western blot image of SLC7A11. (B) Uncropped and full size western blot image of GAPDH. Cropped images are presented in Figure 4D. Cropped bands were indicated in blue boxes. These two images are from two independent SDS PAGE gels.
